# Adherence to 24-h movement guidelines and its associations with dietary behavior and mental health among university students from five ASEAN countries

**DOI:** 10.1186/s12889-025-22643-1

**Published:** 2025-04-30

**Authors:** Supa Pengpid, Karl Peltzer

**Affiliations:** 1 Department of Health Education and Behavioral Sciences, Faculty of Public Health, Mahidol University, Bangkok, Thailand; 2 Department of Public Health, Sefako Makgatho Health Sciences University, Pretoria, South Africa; 3 Department of Healthcare Administration, College of Medical and Health Science, Asia University, Taichung, Taiwan; 4 Department of Psychology, University of the Free State, Bloemfontein, South Africa; 5 Department of Psychology, College of Medical and Health Science, Asia University, Taichung, Taiwan

**Keywords:** Dietary behaviour, Mental health, University students, Southeast Asia, Movement guidelines

## Abstract

**Objective:**

Despite growing evidence linking 24-h movement behaviors to health outcomes, there is limited research examining these relationships among university students in Southeast Asia. Therefore, the purpose of this study was to examine the prevalence and associations of adherence to 24-h movement guidelines (HMG) with dietary behaviour and mental health among university students in ASEAN.

**Methods:**

A multi-center cross-sectional survey was conducted in 2015 included 3223 university students from five ASEAN nations—Indonesia, Malaysia, Myanmar, Thailand, and Vietnam—aged 18 to 30 years, selected by stratified random sampling. Established measures included demographics, social support, perceived stress, self-rated health status, body mass index, six dietary behaviours, eight mental health outcomes, and adherence to 24-h movement guidelines (24-HMG) was assessed based on self-reported physical activity (≥ 150 min/week), sedentary time (≤ 480 min/day), screen time (≤ 180 min/day), and sleep duration (7–9 h). The relationship between dietary behaviours, mental health indicators and meeting the 24-HMG number was investigated using logistic regression models, adjusted for relevant confounders.

**Results:**

Among 3,223 participants, 11.7% met all three 24-h movement guidelines, while 13.1% met none, and 37.6% met either one or two guidelines. Adherence to all guidelines was higher among males, participants aged 22–30 years, and those from lower-income countries (Indonesia, Myanmar, and Vietnam). Multiple logistic regression analyses, adjusted for relevant confounders, revealed that meeting more movement guidelines was associated with: Increased odds of healthy dietary behaviors (fruit and vegetable intake: Adjusted Odds Ratio-AOR: 1.96, 95% Confidence intervals-CI: 1.35–2.83; breakfast intake: AOR: 2.48, 95% CI: 1.84–3.34; meal frequency: AOR: 1.83, 95% CI: 1.36–2.45; low soft drink intake: AOR: 2.20, 95% CI: 1.54–3.14; high sugared coffee or tea intake: AOR: 0.39, 95% CI: 0.24–0.61; low fast food intake: AOR: 1.46, 95% CI: 1.08–1.96; and low snacking: AOR: 2.71, 95% CI: 2.00–3.66), and Decreased odds of mental health issues (depressive symptoms: AOR: 0.43, 95% CI: 0.26–0.70; suicidal behaviour: AOR: 0.66, 95% CI: 0.47–0.93; pathological internet use: AOR: 0.60, 95% CI: 0.45–0.81; hazardous or harmful alcohol use: AOR: 0.44, 95% CI: 0.29–0.66; illicit drug use: AOR: 0.38, 95% CI: 0.20–0.73; and had poorer sleep quality: AOR: 0.33, 95% CI: 0.16–0.68). No significant associations were found between guideline adherence and PTSD symptoms or tobacco use (p > 0.05).

**Conclusions:**

This is the first study to look at the prevalence, correlates, and relationships between 24-HMG adherence and dietary behaviour and mental health outcomes among university students in ASEAN. This pioneering study among ASEAN university students demonstrates that adherence to 24-h movement guidelines is associated with better dietary behaviours and mental health outcomes in a dose–response manner. Future university health policies should incorporate 24-h movement guidelines into existing health promotion strategies, particularly in resource-limited settings.

## Introduction

Previous research has demonstrated that individual movement behaviours, including adequate physical activity [1], low sedentary time [2, 3], and sufficient sleep duration [4] impact positively on health in adults. Changes in the length of one movement behaviour over the course of a day will change the length of another. Consequently, recent studies have examined the ways in which these behaviours interact to affect health outcomes [5, 6]. Subsequently, for adults eighteen years of age and above, the Canadian Society for Exercise Physiology (CSEP) has suggested that a 24-h movement guideline (HMG) should incorporate physical activity, sedentary behaviour, and sleep as essential elements. The first 24-HMG for adults 18–64 years old were released by the CSEP in October 2020 [6]. Based on the time use of the day, a research framework was proposed that integrates sleep, sedentary time, and physical activity time [7]. This combination is thought to provide more reliable information on health behaviour change [8, 9]. According to Ross et al. [6], there are three main recommendations for adults in 24-HMG: increase physical activity, decrease sedentary time, and get a good night's sleep. Some previous studies among university students investigated the association between 24-HMG and a single dietary behaviour or a single mental health outcome. There are apparently no studies on the prevalence, and associations of multiple dietary behaviour and mental health outcomes of 24-HMG among university students, which prompted the study.

Dietary behaviours are influenced by social-cultural and familial factors and differ by region and time [10]. University students are especially vulnerable to nutritional imbalances because they have more freedom to choose how they want to live [11]. This may lead to a higher intake of high consumption of energy-dense snack foods, ultra-processed foods, fast foods, and sugar-sweetened beverages [12, 13], and a low daily intake of fruits and vegetables [14, 15], skipping breakfast [16] and skipping meals [17]. However, healthy dietary behaviours, such as adequate fruit and vegetable (FV) consumption [18], regular breakfast [19], reduced intake of ultra-processed foods, fast foods, unhealthy snacks, and sugar-sweetened beverages [20], and having regular meals [17] are essential for both physical and mental health. Existing research on 24-HMG and dietary behaviour found among university students in China meeting all three guidelines was associated with a greater FV intake compared to not meeting either guideline [11]. In comparison to those who did not follow the three 24-HMG, Spanish teenagers who met all three of the 24-HMG had greater adherence to the Mediterranean diet, were more likely to eat FV once a day, and were less likely to eat pastries or commercially baked goods for breakfast as well as sweets and candies multiple times a day [21].

Furthermore, university students experience a variety of pressures and challenges as they move from adolescence to adulthood [22, 23], which increases their vulnerability to psychological issues, such as depressive symptoms. For instance, participants in a study of university students from 26 low-, middle-, and high-income nations reported a prevalence of 24.0% for moderate depression and 12.8% for severe depression [24]. Thirty-one percent of college students from nineteen colleges spread across eight countries—Australia, Belgium, Germany, Mexico, Northern Ireland, South Africa, Spain, and the United States—screened positive for at least one 12-month mental illness, the most common of which being major depression, mania/hypomania, panic disorder, generalized anxiety disorder, alcohol use disorder, and substance use disorder [25]. Existing studies among university students from China, showed following fewer 24-HMG was linked to depressive symptoms [22], severe anxiety symptoms in China [26], and poorer mental health [27]. Studies from university students in North America showed that adherence to concurrent adherence to all three guidelines were associated with significantly reduced odds of suicidal ideation but not suicide planning [28] and meeting all movement guidelines was associated with lower depressive symptoms [29]. Among adolescents without smartphone addiction engaged in less sedentary behaviour (SB) and engaged in more moderate-to-vigorous physical activity (MVPA) and sleep duration than adolescents with smartphone addiction [30]. A recent systematic review [31] found that positive effects were most frequently seen for mental health and wellbeing indicators across the lifespan when all three 24-HMG criteria were satisfied.

**Fig. 1:**
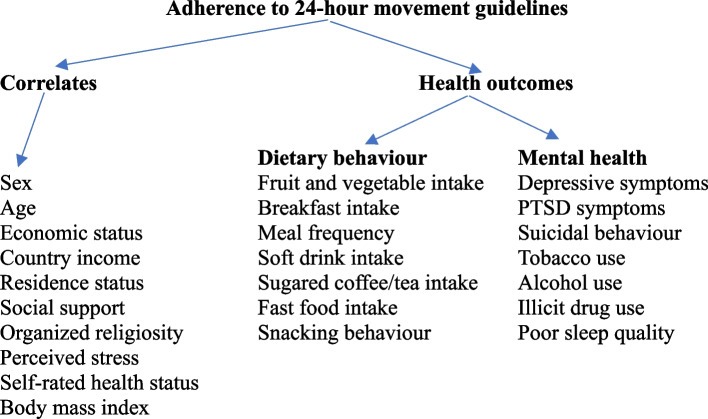
Conceptual framework of 24-h movement behaviours

However, it appears that previous investigations among university students found a low rate of adherence to meeting three of the 24-HMG, e.g., in China 27.8% [22] and 31.72% [32], and in Canada, only 9.9% of students were currently achieving all components of the 24-HMG [33]. Canadian university students most commonly adhered to MVPA (61.1%), followed by sleep (59.7%), sedentary and screen time guidelines (56.3% and 36.2%, respectively) [33], while among Chinese university students adhering to SB guidelines was the highest (70.6%), followed by sleep (70.3%) and MVPA guidelines (48.6%) [22]. Correlates of achieving all components of the 24-HMG among university students included women, high mental well-being, little or no mental distress [33] and adherence to at least one 24-HMG, male students, greater body mass index (BMI), higher perceived family influence and greater number of friends [26]. Among Thai adults, the lowest odds for meeting the 24-h guidelines were found among males, those living in urban areas, unemployed, and those with low education level [8]. Owing to a lack of information, our goals were to: (1) find out how common it is for university students to meet 24-HMG and its correlates; and (2) assess the relationship between adherence to 24-HMG and seven dietary behaviour outcomes (fruit and vegetable intake, breakfast consumption, meal frequency, soft drink intake, sugared coffee or tea intake, fast food consumption and snacking) and eight mental health outcomes (depressive symptoms, PTSD, suicidal behaviour, pathological internet use, tobacco use, hazardous or harmful alcohol use, illicit drug use and sleep quality) in university students in Southeast Asia. (see conceptual framework: Fig. 1).

## Methods

### Sample and procedure

A cross-sectionally self-administered survey in 2015 included 3223 university students (median age 20 years, interquartile range = 3) with complete 24-HMG measurements from 5 ASEAN countries: in Indonesia (in Yogyakarta), Malaysia (in Kuala Lumpur), Myanmar (in Yangon), Thailand (in Maha Sarakham) and Vietnam (in Hanoi). Previous descriptions of methodological procedures can be found here [34]. To put it briefly, deliberate sampling was used to choose one university per nation. Undergraduate students at each university were chosen at random for anthropometric measurements and a self-administered questionnaire survey using a stratified random sampling technique. In each study country, investigators aimed for a total sample of 800 university students, 400 males and 400 females, aged 18–30 years. However, some of the participating locations did not meet the recruitment target. Students who took part signed informed consent forms. All countries had over 90% participation rates, with the exception of Indonesia (69%), and Myanmar (73%). Ethics approvals were obtained from all participating universities: “University of Malaya Medical Ethics committee (MECID 201412–905)”, “Research and Ethical Committee of University of Medicine 1”, “Committee for Research Ethics (Social Sciences) of Mahidol University (MU-SSIRB 2015/116(B2)”, “Committee of Research Ethics of Hanoi School of Public Health”, and “Research Ethics Committee, Faculty of Medicine and Health Sciences, Universitas Muhammadiyah Yogyakarta.” All methods were carried out in accordance with relevant guidelines and regulations and have been performed in accordance with the Declaration of Helsinki.

### Measures

The 24-HMG requirements were: 150 min or more of physical activity per week; 480 min or less of sedentary time; 180 min or less of screen time; and 7–9 h of sleep for those aged 18 to 64 [6].

"Low, moderate, and high physical activity [35]; the latter two representing MVPA was the classification used to measure physical activity using the"International Physical Activity Questionnaire (IPAQ) short-form questionnaire"

The IPAQ [35] provided data on sedentary behaviour. It asked,"How much time did you spend sitting on weekdays over the last seven days? Incorporate time spent on coursework, at work, at home, and during free time. This could involve reading, visiting friends, working at a desk, or lounging or sitting down to watch TV."How much time did you spend sitting on a weekday during the last seven days?"(daily hours/daily minutes).

Furthermore, the question"How many hours do you normally spend in a day on the internet?"was used to gauge screen time. a) during work or academic hours, and b) during leisure or personal hours.

The question"On average, how many hours of sleep do you get in a 24 h period? (hours)"was used to measure the length of sleep [36].

### Dietary behaviour outcomes

Two questions were used to gauge intake of fruits and vegetables: 1)"How many servings [one standard serving = 80 g] of fruit do you eat on a typical day?"and 2)"What is your daily intake of vegetables, measured in servings [one standard serving = 80 g]?"[37]."Five or more servings a day"was the definition of an adequate intake of fruits and vegetables [38].

"How often do you eat breakfast?"was the item used to gauge breakfast consumption. ("Almost daily, occasionally, infrequently, or never").

The question, “How many meals do you eat each day?” was used to calculate meal intake. (coded 0 = < 2 times/day and 1 = 3 or more times/day) [36].

The question,"During the past 30 days, how many times per day did you usually drink carbonated soft drinks (do not include diet soft drinks)?"was used to measure"soft drink consumption."Responses were categorized as follows: 1 = I did not consume carbonated soft drinks for the previous thirty days; or = Less than one time per day; 0 = One time per day; or 2 times per day; or 3 times per day; or 4 times per day; or 5 or more times per day.

Sugared coffee or tea consumption was assessed with the question, “How often do you have sugared coffee or tea?” Responses ranged from 1 = more than once a day to 6 = never, and coded as 1 = “more than once a day or once a day” and 0 = ”3–6 times a week, 1–2 times a week, rarely or never.”

The question,"During the past 7 days, on how many days did you eat food from a fast-food restaurant?"was used to gauge intake of fast food. (with 0 = 1–7 days and 1 = 0 days coded).

Among the questions about snacking was"How many between-meal snacks do you eat each day?"(coded 1 as 0 or 1 and 0 as 2 or more each day) [36].

### Mental health outcomes

The"Centers for Epidemiologic Studies Depression Scale (CES-D- 10)"was used to measure depressive symptoms from the previous week (scores of 15 or higher) [39]. Ten questions make up the CESD- 10, such as"I was bothered by things that usually don't bother me."Total scores varied from 0 to 30, with higher scores indicating higher depressive symptom scores [39]. Response options ranged from 0 ="Rarely (< 1 day)"to 3 ="Most (5–7 days)". In this sample, the CES-D- 10's Cronbach's alpha was 0.74. The psychometric qualities of the CES-D- 10 have been described in this study population of university students in low- and middle-income countries [40], and it has been demonstrated that the CES-D- 10 can accurately identify clinical depression in adolescent and adult samples [39].

A seven-item short screening scale for DSM-IV PTSD was used to measure symptoms of PTSD from the previous month (Yes, No). Those who answered"yes"to at least four of the questions were deemed to have a positive screen for PTSD [41], identifying cases of PTSD with a sensitivity of 78% and specificity of 97% [42]. The screen was scored by adding up the positive responses, with scores ranging from 0 to 7. The 7-item short screening scale for DSM-IV PTSD had a Cronbach's alpha of 0.77.

The concepts of ever suicidal ideation, planning, and attempt were taken from an Osman et al. study [43].

Using the"Young Diagnostic Questionnaire for Internet Addiction (YDQ),"pathological internet use was quantified [44] (0.70 Cronbach's alpha)."Have you repeatedly made unsuccessful attempts to control, cut back, or stop Internet and/or smartphone use?"is one example of a sample question. Eight"yes"or"no"questions are used to evaluate the criteria. The total score can range from 0 to 8, with"pathological users"receiving a score of at least 5 [45].

"Do you currently use one or more of the following tobacco products (cigarettes, snuff, chewing tobacco, cigars, etc.)?"was the only question used to gauge tobacco use. (In agreement, in disagreement).

The"Alcohol Use Disorders Identification Test-Concise (AUDIT-C)"was used to evaluate alcohol consumption that was hazardous or harmful [46] (0.89 was the Cronbach alpha).

"How often have you taken drugs in the past 12 months, other than prescribed by the health care provider?"was the single question used to gauge drug use (in the previous 12 months). The responses were categorized as 1 = 1 or more times and 0 = 0 times.

"Severe or extreme having a problem with sleeping, such as falling asleep, waking up frequently during the night, or waking up too early in the morning in the past 30 days?"was the definition of poor sleep quality.

### Confounding variables

Age, sex, country income, subjective wealth, and residency status were among the sociodemographic variables. In 2015, higher income countries (upper middle-income) included Malaysia and Thailand and lower income countries (lower middle-income) included Indonesia, Myanmar, and Vietnam."Family background as Wealthy (within the highest 25% in your country in terms of wealth), quite well off (within the 50–75% range for your country), not very well off (within the 25–50% range for your country), or quite poor (within the lowest 25% in your country in terms of wealth”)"was the scale used to determine a person's wealth status [36]. We then defined low wealth status as being relatively poor or not very well off, and high wealth status as being wealthy or well off. The inquiry,"What is your current residence?"was used to determine residence status. Three options were available for responding: (1) on campus, (2) off campus (by yourself), and (3) off campus (with parents/guardians).

Three questions from the"Social Support Questionnaire (SSQ)"were used to gather social support data [47]."I feel like there is no one with whom I can share my most private concerns,"for instance. The answers varied from 1 (wholly true) to 4 (wholly false). Higher scores indicate higher levels of social support. The three items'scores were added up to produce a score range of 3 to 12. The total scores were divided into two categories: low social support (3–8) and high social support (9–12). In this sample, the 3-item SSQ index's Cronbach's alpha was 0.94.

One dimension of the"Duke University Religion Index"[48] was used to measure organized religiosity:"How often do you attend church, temple, or other religious meetings?"(categorized as high = once a week or more than once a week, medium = a few times a year or a few times a month, and low = never or once a year or less).

Three items, such as"I often feel helpless in dealing with the problems of life,"were used to measure perceived stress [49]. A 5-point Likert scale, with 1 denoting"strongly disagree"and 5 denoting"strongly agree,"is used to rate each item. With Cronbach's α of 0.77. By employing the median as a cut point, the scale was made dichotomous.

Questionnaire on one's own health status:"Overall, how would you rate your health? Excellent, very good, good, fair, or poor?""Fair or poor health"was the definition of"poor health."

"With standard anthropometric measurements,"[50] the BMI was calculated. Asian criteria measuring BMI at least 25.0 kg/m^2^ were used to define obesity [51].

### Data analysis

Descriptive statistics were used to describe the sample characteristics, and Chi-square tests and student-t tests were used to test for differences in proportions and means, respectively. Unadjusted and adjusted logistic regression analyses were used to assess the associations between 24-HMG categories (0, 1, 2, and 3, with 0 24-HMG as reference) with six dietary behaviour outcomes (fruit and vegetable intake, breakfast consumption, meal frequency, soft drink intake, fast food consumption and snacking) eight mental health outcomes (depressive symptoms, PTSD, suicidal behaviour, pathological internet use, tobacco use, hazardous or harmful alcohol use, illicit drug use and sleep quality) was assessed using multivariable logistic regression, after adjusting for age group, sex, subjective economic status, residence status, country, perceived stress, social support, organized religiosity, self-rated health status, and body mass index. Covariates were included based on previous research, including BMI [22, 52] stress, self-rated health [53], sociodemographic factors [11, 22] on the associations of PA, SB, and sleep with health in university students or adults. Furthermore, unconditional adjusted logistic regression was used to estimate the correlates of 24-HMG and its components. Results from the logistic regression analyses are reported as odds ratios (ORs) with 95% confidence intervals (CIs). Missing values (< 5% on key variables) were eliminated from the analysis as there was no evidence of collinearity. p less than 0.05 was deemed significant. Data analysis was conducted with “STATA software version 15.0 (Stata Corporation, College Station, TX, USA)”. Country was used as the primary sample unit for survey analysis in STATA in order to achieve accurate CIs due to the data's clustered structure. The study did not include any missing data.

## Results

### Descriptive statistics of study variables

In total 3223 university students from five ASEAN nations—Indonesia, Malaysia, Myanmar, Thailand, and Vietnam—aged 18 to 30 made up the sample; 62.6% of them were women.

The percentages of participants who adhered to none, one, and two movement guidelines were 13.1%, 37.6%, and 37.6%, respectively, whereas 11.7% of participants met all three guidelines. Specifically, 45.8% of participants met the individual movement guidelines for MVPA, 47.2% for SB, and 50.1% for sleep. Adherence to 24-HMG levels differed by country, sex, age, subjective economic status, country income, residence status, social support, organized religiosity and perceived stress. More details of the study sample are presented in Table 1.

**Table 1:** Sample and 24-h movement guidelines (HMG) levels, university students

Variable	Sample	24_HMG levels				
	Total	0	1	2	3	*p*-value
	N (%)	%	%	%	%	
All	3223	13.1	37.6	37.6	11.7	
Country Indonesia Malaysia Myanmar Thailand Vietnam	229 (7.1) 1023 (31.7) 370 (11.5) 785 (24.4) 816 (25.3)	24.9 12.1 8.9 20.6 5.6	56.3 36.3 36.8 46.6 25.7	17.0 39.6 48.9 26.5 46.3	1.7 12.0 5.4 6.2 22.3	< 0.001
Sex Female Male	2018 (62.6) 1205 (37.4)	15.1 9.7	39.2 34.9	36.2 39.8	9.4 15.6	< 0.001
Age 18–19 20–21 22–30	964 (29.9) 1402 (43.5) 857 (26.6)	15.8 12.6 10.9	42.3 36.2 34.5	33.3 40.5 37.6	8.6 10.6 17.0	< 0.001
Subjective economic status Low High	2155 (66.9) 1068 (33.1)	12.2 14.9	36.6 39.6	38.1 36.5	13.1 9.0	< 0.001
Country income Lower Higher	1808 (56.1) 1415 (43.9)	9.6 15.8	33.6 40.8	42.3 33.9	14.6 9.5	< 0.001
Residence status Live with parents/guardians Live away from parents	636 (19.7) 2586 (80.3)	10.2 13.8	32.7 38.8	46.5 35.3	10.5 12.0	< 0.001
Social support Low High	1522 (47.4) 1692 (52.6)	13.0 13.2	40.8 34.6	35.5 39.4	10.6 12.8	0.002
Organized religiosity Low Medium High	923 (28.7) 1564 (48.6) 729 (22.7)	10.8 14.1 13.9	31.2 39.8 40.9	41.4 36.4 35.0	16.6 9.6 10.3	< 0.001
Perceived stress Low High	1277 (39.7) 1937 (60.3)	12.5 13.5	35.2 39.3	39.3 36.2	13.0 10.9	0.030
Self-rated health status Moderate/high Low	2403 (74.6) 819 (25.4)	12.6 14.5	38.4 35.3	37.3 38.2	11.7 12.0	0.323
Body mass index < 25 kg/m^2^ ≥ 25 kg/m^2^	2676 (87.4) 385 (12.6)	13.0 15.6	37.1 41.0	37.9 34.5	12.0 8.8	0.071

### Associations with adherence to 24-h movement guidelines

Male sex, aged 22 to 30 years, and living in a lower income country (Indonesia, Myanmar, and Vietnam) were associated with adhering to all three 24-HMG. Male students adhered to the MVPA and SB individual movement guidelines. Older students were more likely to adhere to all three individual 24-HMG. Higher subjective wealth status decreased the odds of adhering to the MVPA and increased the odds of adhering to the SB individual movement guidelines. Living in a lower middle-income country increased the odds and living away from parents or guardians decreased the odds of adhering to SB and sleep guidelines (see Table 2).

**Table 2:** Correlates of adherence to 24-h movement guidelines, university students, ASEAN, *N* = 3223

Variable	Physical activity	Sedentary behaviour	Sleep	All 3 guidelines
	AOR (95% CI)^a^	AOR (95% CI)^a^	AOR (95% CI)^a^	AOR (95% CI)^a^
Sex Female Male	1 (Reference) 1.53 (1.31 to 1.79)***	1 (Reference) 1.23 (1.05 to 1.43)*	1 (Reference) 1.06 (0.90 to 1.24)	1 (Reference) 1.65 (1.31 to 2.08)***
Age 18–19 20–21 22–30	1 (Reference) 1.31 (1.10 to 1.56)** 1.27 (1.04 to 1.54)*	1 (Reference) 1.13 (0.95 to 1.34) 1.26 (1.04 to 1.54)*	1 (Reference) 1.07 (0.90 to 1.28) 1.37 (1.12 to 1.67)***	1 (Reference) 1.19 (0.89 to 1.59) 1.76 (1.30 to 2.39)***
Subjective economic status Low High	1 (Reference) 0.72 (0.61 to 0.84)***	1 (Reference) 1.27 (1.08 to 1.49)**	1 (Reference) 0.90 (0.76 to 1.06)	1 (Reference) 0.79 (0.61 to 1.04)
Country income Higher Lower	1 (Reference) 0.85 (0.71 to 1.02)	1 (Reference) 1.73 (1.45 to 2.07)***	1 (Reference) 2.02 (1.69 to 2.41)***	1 (Reference) 1.35 (1.03 to 1.78)*
Residence status Live with parents/guardians Live away from parents	1 (Reference) 1.06 (0.86 to 1.31)	1 (Reference) 0.73 (0.60 to 0.90)**	1 (Reference) 0.69 (0.55 to 0.85)***	1 (Reference) 1.22 (0.89 to 1.68)
Social support Low High	1 (Reference) 1.17 (1.01 to 1.35)*	1 (Reference) 1.02 (0.88 to 1.18)	1 (Reference) 1.07 (0.92 to 1.24)	1 (Reference) 1.14 (0.91 to 1.44)
Organized religiosity Low Medium High	1 (Reference) 0.80 (0.66 to 0.96)* 0.85 (0.68 to 1.07)	1 (Reference) 1.14 (0.95 to 1.38) 1.45 (1.16 to 1.82)***	1 (Reference) 0.79 (0.66 to 0.96)* 0.66 (0.52 to 0.83)***	1 (Reference) 0.76 (0.58 to 1.00) 0.79 (0.56 to 1.11)
Perceived stress Low High	1 (Reference) 1.09 (0.93 to 1.27)	1 (Reference) 0.85 (0.73 to 0.99)*	1 (Reference) 1.15 (0.98 to 1.34)	1 (Reference) 0.96 (0.76 to 1.21)
Self-rated health status Low Moderate/high	1 (Reference) 1.12 (0.94 to 1.33)	1 (Reference) 1.11 (0.93 to 1.32)	1 (Reference) 0.94 (0.79 to 1.12)	1 (Reference) 1.08 (0.83 to 1.41)
Body mass index < 25 kg/m^2^ ≥ 25 kg/m^2^	1 (Reference) 0.86 (0.68 to 1.07)	1 (Reference) 0.88 (0.70 to 1.10)	1 (Reference) 0.76 (0.61 to 0.96)*	1 (Reference) 0.70 (0.48 to 1.03)

*AOR* Adjusted Odds Ratio, *CI* Confidence Interval

^a^Adjusted for all variables in the table

### Associations between dietary behaviour outcomes and following 24-h movement guidelines

In adjusted analysis, compared with meeting none of the 24-HMG, participants who met a higher number of recommendations in a dose–response fashion were more likely to report adequate fruit and vegetable intake, had almost always breakfast, three or more meals a day, had no or less than 1 soft drink per day in the past 30 days, had less than daily sugared coffee or tea, had less than 1 day fast food intake in the past week, and had none or 1 snack in a day (see Table 3).

**Table 3:** Associations between adhering to 24-h movement guidelines (HMG) and dietary behaviour

**Dietary behaviour**	24-HMG adherence level (0–3)	COR (95% CI)	*p*-value	AOR (95% CI)^a^	*p*-value
**Fruit and vegetable intake (5 +)** (*n* = 765, 24.8%)	0	1 (Reference)		1 (Reference)	
	1	1.42 (1.05 to 1.92)	< 0.001	1.46 (1.07 to 2.00)	0.017
	2	1.72 (1.27 to 2.30)	< 0.001	1.71 (1.25 to 2.33)	< 0.001
	3	2.19 (1.55 to 3.10)	< 0.001	1.96 (1.35 to 2.83)	< 0.001
**Breakfast (almost always)** (*n*** = **1431, 44.4%)	0	1 (Reference)		1 (Reference)	
	1	1.54 (1.22 to 1.95)	< 0.001	1.52 (1.20 to 1.93)	< 0.001
	2	2.13 (1.88 to 2.89)	< 0.001	1.93 (1.52 to 2.45)	< 0.001
	3	2.60 (1.95 to 3.47	< 0.001	2.48 (1.84 to 3.34)	< 0.001
**Meal frequency (≥ 3/day)** (*n* = 1926, 60.0%)	0	1 (Reference)		1 (Reference)	
	1	1.40 (1.12 to 1.75)	0.003	1.36 (1.09 to 1.71)	0.007
	2	1.80 (1.44 to 2.26)	< 0.001	1.64 (1.30 to 2.06)	< 0.001
	3	2.09 (1.57 to 2.79)	< 0.001	1.83 (1.36 to 2.45)	< 0.001
**Soft drink intake (0 or < 1 time/day)** (*n* = 2161, 67.1%)	0	1 (Reference)		1 (Reference)	
	1	0.97 (0.77 to 1.21)	0.764	0.98 (0.77 to 1.26)	0.893
	2	1.30 (1.03 to 1.64)	0.026	1.43 (1.11 to 1.85)	0.006
	3	2.58 (1.86 to 3.58)	< 0.001	2.20 (1.54 to 3.14)	< 0.001
**Sugared coffee or tea (≥ 1 time/day)** (*n* = 593, 18.4%)	0	1 (Reference)		1 (Reference)	
	1	0.69 (0.53 to 0.90)	0.007	0.63 (0.45 to 0.89)	0.008
	2	0.64 (0.49 to 0.84)	< 0.001	0.57 (0.40 to 0.80)	< 0.001
	3	0.52 (0.36 to 0.75)	< 0.001	0.39 (0.24 to 0.61)	< 0..01
**Fast food intake (< 1 day/week)** (*n* = 1466, 45.6%)	0	1 (Reference)		1 (Reference)	
	1	1.08 (0.86 to 1.35)	0.531	1.09 (0.86 to 1.38)	0468
	2	1.21 (0.96 to 1.52)	0.092	1.21 (0.96 to 1.54)	0.111
	3	1.78 (1.35 to 2.36)	< 0.001	1.46 (1.08 to 1.96)	0.013
**Snacking (0 or 1 time/day)** (*n*** = **1621, 51.3%)	0	1 (Reference)		1 (Reference)	
	1	1.55 (1.24 to 1.96)	< 0.001	1.53 (1.21 to 1.94)	< 0.001
	2	2.05 (1.63 to 2.57)	< 0.001	1.93 (1.53 to 2.45)	< 0.001
	3	3.30 (2.46 to 4.42)	< 0.001	2.71 (2.00 to 3.66)	< 0.001

^a^Adjusted for age group, sex, subjective economic status, residence status, country, perceived stress, social support, organized religiosity, self-rated health status, and body mass index; *CI* Confidence Interval, *COR* Crude Odds Ratio, *AOR* Adjusted Odds Ratio

### Associations between mental health outcomes and following 24-h movement guidelines

In adjusted analysis, compared with meeting none of the three 24-HMG, participants who met a higher number of recommendations were less likely to report depressive symptoms, suicidal behaviour, pathological internet use, hazardous or harmful alcohol use, illicit drug use, and had poorer sleep quality, while no significant associations were found for PTSD symptoms and tobacco use (see Table 4).

**Table 4:** Associations between adhering to 24-h movement guidelines (HMG) and mental health

Mental health indicator	24-HMG adherence level (0–3)	COR (95% CI)	*p*-value	AOR (95% CI)^a^	*p*-value
**Depressive symptoms (severe)** (*n* = 347, 10.8%)	0	1 (Reference)		1 (Reference)	
	1	0.79 (0.58 to 1.08)	0.138	0.79 (0.57 to 1.08)	0.132
	2	0.44 (0.31 to 0.61)	< 0.001	0.50 (0.35 to 0.70)	< 0.001
	3	0.37 (0.23 to 0.60)	< 0.001	0.43 (0.26 to 0.70	< 0.001
**PTSD symptoms** (*n* = 792, 24.8%)	0	1 (Reference)		1 (Reference)	
	1	1.02 (0.79 to 1.31)	0.910	1.01 (0.77 to 1.32)	0.947
	2	0.91 (0.70 to 1.18)	0.461	0.95 (0.72 to 1.25)	0.710
	3	1.12 (0.82 to 1.54)	0.481	1.06 (0.75 to 1.49)	0.747
**Suicidal behaviour** (*n* = 375, 11.8%)	0	1 (Reference)		1 (Reference)	
	1	0.88 (0.63 to 1.21)	0.427	0.86 (0.62 to 1.20)	0.383
	2	0.72 (0.51 to 0.99)	0.049	0.66 (0.47 to 0.93)	0.019
	3	0.75 (0.49 to 1.14)	0.176	0.70 (0.45 to 1.08)	0.107
**Pathological internet use** (*n* = 1155, 36.3%)	0	1 (Reference)		1 (Reference)	
	1	0.76 (0.60 to 0.95)	0.016	0.75 (0.59 to 0.94)	0.014
	2	0.58 (0.46 to 0.73)	< 0.001	0.61 (0.48 to 0.77)	< 0.001
	3	0.60 (0.45 to 0.80)	< 0.001	0.60 (0.45 to 0.81)	< 0.001
**Tobacco use (past month)** (*n* = 76, 2.4%)	0	1 (Reference)		1 (Reference)	
	1	1.19 (0.44 to 3.24)	0.730	0.85 (0.30 to 2.42)	0.762
	2	0.84 (0.29 to 2.38)	0.736	0.57 (0.19 to 1.73)	0.320
	3	1.57 (0.50 to 5.00)	0.442	1.22 (0.37 to 4.05)	0.741
**Hazardous or harmful alcohol use** (*n* = 509, 15.9%)	0	1 (Reference)		1 (Reference)	
	1	0.72 (0.55 to 0.94)	0.016	0.76 (0.57 to 1.01)	0.055
	2	0.43 (0.32 to 0.56)	< 0.001	0.45 (0.33 to 0.61)	< 0.001
	3	0.41 (0.28 to 0.61)	< 0.001	0.44 (0.29 to 0.66)	< 0.001
**Illicit drug use in the past 12 months** (*n* = 256, 8.3%)	0	1 (Reference)		1 (Reference)	
	1	0.65 (0.45 to 0.94)	0.023	0.62 (0.42 to 0.92)	0.017
	2	0.73 (0.51 to 1.05)	0.091	0.69 (0.47 to 1.03)	0.067
	3	0.30 (0.16 to 0.56)	< 0.001	0.38 (0.20 to 0.73)	< 0.001
**Poor sleep quality (severe/extreme)** (n = 147, 4.6%)	0	1 (Reference)		1 (Reference)	
	1	0.51 (0.33 to 0.80)	0.003	0.51 (0.32 to 0.80)	0.004
	2	0.45 (0.28 to 0.70)	< 0.001	0.49 (0.31 to 0.78)	0.003
	3	0.33 (0.17 to 0.86)	0.002	0.33 (0.16 to 0.68)	0.003

*CI* Confidence Interval, *PTSD* Posttraumatic Stress Disorder, *COR* Crude Odds Ratio, *AOR* Adjusted Odds Ratio

^a^Adjusted for age group, sex, subjective economic status, residence status, country, perceived stress, social support, organized religiosity, self-rated health status, and body mass index

## Discussion

The first data on the prevalence, correlates, and relationships between 24-HMG adherence and six dietary behaviours and eight mental health outcomes in ASEAN university students are presented by this study. Only 11.7% of participants met all three 24-HMG, according to the results, whereas 13.1%, 37.6%, and 37.6%, respectively, adhered to none, one, and two movement guidelines. Participants who were male, aged 22–30 years and those from lower resourced countries were more likely to meet all three 24-HMG. Compared with meeting none of the 24-HMG, participants who met a higher number of recommendations in a dose–response fashion were more likely to report healthy dietary behaviours (adequate fruit and vegetable intake, had almost always breakfast, had three or more meals a day, had no or less than one soft drink per day in the past 30 days, had less than daily sugared coffee or tea, had less than one day fast food intake in the past week, and had none or one snack in a day), and less likely to report poor mental health issues (depressive symptoms, suicidal behaviour, pathological internet use, hazardous or harmful alcohol use, illicit drug use, and poorer sleep quality), while no significant associations were found for PTSD symptoms and tobacco use.

Regarding the proportion of guideline adherence, the percentage of ASEAN university students meeting all three 24-HMG (11.7%) was lower than among university students in China (27.8%) [22], and (31.7%) [32], but higher than among Canadian university students (9.9%) [33]. In terms of 24-HMG correlates, consistent with previous findings in China [26], male sex was associated with being more likely to meet all three 24-HMG and individual 24-HMG. Nevertheless, results stress the need for 24-HMG health promotion activities among ASEAN university students by taking into account some found sociodemographic differences in adherence to 24-HMG. For example, students from a high family wealth background were less likely to adhere to MVPA and were more likely to adhere to SB individual movement guidelines.

Similar to a study among university students in China [22], among the three individual movement guidelines, adhering to MVPA guidelines was the lowest, followed by sleep and SB guidelines in this study.

Consistent with studies among university students in China [11] and adolescents in Spain [21] meeting all three guidelines was associated with healthy dietary behaviour (a greater fruit and vegetable intake, regular breakfast and meals, and less fast foods, and sugar-sweetened beverages) compared to adhering to fewer guidelines. Several reviews have shown that individual MVPA, SB, and sleep have beneficial effects on mental well-being. For example, studies using observational data substantiate a non-linear relationship between adults who get the recommended 7–9 h of sleep per day and their highest intakes of fruit and vegetables (FV). FV consumption is impacted by sleep disruption through both homeostatic and non-homeostatic mechanisms [54]. In a multi-country study among adolescents SB was associated with fast food and soft drink consumption [55]. Among youth SB and unhealthy dietary behaviour (such as the consumption of energydense food products) have repeatedly been found to cluster [56], and among adults, screen time (such as watching TV and using a computer) was negatively correlated with eating fruit and vegetables and positively correlated with snacking and the consumption of sugar-sweetened beverages. Unhealthy eating habits and sedentary behaviour may co-occur or cluster together as a result of shared environmental cues [57]. Among adults in Bazil, physical activity was positively associated with healthier eating habits [58].

In line with several previous studies among university students [22, 27, 28, 30], this study found an association between higher adherence to 24-HMG and poorer mental health (depressive symptoms, suicidal behaviour, pathological internet use). In the case of suicidal behaviour, compared to adhering to zero 24-HMG, adhering to two 24-HMG reduced the odds of suicidal behaviour significantly but not adhering to three 24-HMG. In additional analysis on the association between individual adherence to 24-HMG, it was found that only SB reduced and not MVPA and sleep significantly reduced suicidal behaviour. While a study among university students in China [26] found an association between higher adherence to 24-HMG and lower severe anxiety, our study did not find a significant association between higher adherence to 24-HMG and lower PTSD symptoms. Previous research among adolescents found mixed results regarding the association between adherence to 24-HMG and substance [59], while this study found that students with a higher number of 24-HMG recommendations were more less likely to report hazardous or harmful alcohol use, and illicit drug use, but there was no significant association with tobacco use. Finally, the association between a higher number of 24-HMG recommendations and poorer sleep quality, may be explained by significant negative contribution of sleep duration of 7–9 h, positive contribution of MVPA and no contribution by SB. Several reviews have shown that individual MVPA, SB, and sleep have beneficial effects on mental well-being. For example, MVPA may reduce depressive symptoms [60] and improve PTSD symptoms and quality of life in adults with PTSD [61]. The risk of depression may rise with mentally passive sedentary behaviours like watching television [62, 63] and SB is associated with PTSD [64]. Research has shown that prolonged wakefulness, short sleep durations, and/or nighttime awakenings have a negative impact on how people feel emotionally [65]. Evidence for the potential of physical activity interventions to improve undergraduate students'mental health has been reviewed recently [64].

Our study's results confirm the link between university students'dietary behaviour and mental health outcomes and following movement guidelines. A dose–response gradient was observed between improved dietary behaviour and mental health indicators and an increase in the number of recommendations fulfilled. Future studies are required to explore the precise mechanisms through which the combination of movement behaviours improve dietary behaviour and mental health, which will help to inform the creation of more potent health promotion programmes. Colleges should evaluate a dual or multiple-pronged approach that simultaneously promotes healthier movement behaviours, a healthy diet, and mental wellbeing while taking student characteristics into account, given the plausible bi- or tri-directional associations between movement behaviors, dietary behaviors, and mental health [29].

### Study limitations and strength

The cross-sectional survey design, the restriction to university students, and the deliberately chosen universities were among the study's limitations. As a result, we were unable to generalize the findings and ascertain the direction of the relationship between 24-HMG and dietary behaviour and mental health outcomes. Future research should consider longitudinal designs to better understand the temporal relationships between 24-HMG adherence and health outcomes. Self-reporting was used to gather the data, which could have led to biased answers. Additionally, screening instruments were used to evaluate PTSD and depression symptoms rather than conducting a clinical psychiatric interview. Furthermore, some of the outcome measures, e.g., sleep quality, were only assessed with single items and future research should include multiple item measures. In addition, the study was conducted in 2015, which is prior to the Canadian 24-HMG for adults in 2020, and also prior to issuing 24-HMG in the study countries, e.g., in Thailand 24-HMG was issued in 2017 [65], which makes us unable to estimate more recent effects of the 24-HMG. The study's strength was the use of standard evaluation instruments for college students across five nations. While the study includes multiple confounding variables, future studies could benefit from including academic performance metrics and seasonal variations in behaviour patterns, as these factors might influence both movement behaviours and health outcomes among university students. Furthermore, the estimates of servings of fruit and vegetables could have been enhanced by pictures or food models to help with the estimation.

## Conclusions

This is the first study to look at the prevalence, correlates, and relationships between 24-HMG adherence and multiple dietary behaviours and multiple mental health outcomes among university students in Southeast Asia. Adhering to a higher number of 24-HMG was associated with seven healthy dietary behaviours and six mental health outcomes. The results highlight how critical it is to encourage ASEAN university students to engage in healthy movement behaviours. Future dietary and mental health interventions for ASEAN university students should focus on improving their overall movement behaviours while taking demographic differences into account. Future university health policies should incorporate 24-h movement guidelines into existing health promotion strategies, particularly in resource-limited settings.

## Data Availability

Since only the researchers working on the project would be able to access the information participants provided, the data from this study will not be made available to the general public.

## Abbreviations

ASEAN: Association of Southeast Asian Nations
AUDIT-C: Alcohol Use Disorders Identification Test-Concise
BMI: Body mass index
CSEP: Canadian Society for Exercise Physiology
CES-D- 10: 10 Item Center for Epidemiologic Studies Depression Scale
FV: Fruit and vegetables
IPAQ: International Physical Activity Questionnaire
IQR: Interquartile range
MVPA: Moderate and vigorous physical activity
PTSD: Posttraumatic stress disorder
SB: Sedentary behavior
SSQ: Social Support Questionnaire
24-HMG: 24-Hour movement guidelines
YDQ: Young Diagnostic Questionnaire for Internet Addiction
